# Digitization of imaging plates from Guinier powder X-ray diffraction cameras

**DOI:** 10.1107/S160057672200677X

**Published:** 2022-08-24

**Authors:** Jamal Nasir, Nils Steinbrück, Ke Xu, Bernward Engelen, Jörn Schmedt auf der Günne

**Affiliations:** a Inorganic Materials Chemistry and Center of Micro- and Nanochemistry and Engineering (Cμ), Department of Chemistry and Biology, Faculty IV: School of Science and Technology, University of Siegen, Adolf-Reichwein-Straße 2, Siegen, Nordrhein-Westfalen D-57076, Germany; b Inorganic Chemistry I, Department of Chemistry and Biology, Faculty IV: School of Science and Technology, University of Siegen, Adolf-Reichwein-Straße 2, Siegen, Nordrhein-Westfalen D-57076, Germany; Australian Synchrotron, ANSTO, Australia

**Keywords:** *IPreader* software, Guinier cameras, imaging plates (IPs), diffraction pattern conversion into data columns, powder X-ray diffraction, data processing, Guinier method

## Abstract

A Guinier camera equipped with an imaging plate is used to investigate and eliminate the sources of instrumental errors affecting the quality of the obtained scanned Guinier data. A program with a graphical user interface is presented which converts the data of the scanned images into different standard file formats for powder X-ray patterns containing intensities, their standard deviations and the diffraction angles. The program also allows for manual and automatic correction of the 2θ scale against a known reference material. It is shown using LaB_6_ that the exported X-ray diffraction patterns provide a 2θ scale reproducible enough to allow for averaging diffractograms obtained from different exposures of the imaging plate for the same sample. As shown on a mixture of NaCl and sodalite, the quality of the produced data is sufficient for Rietveld refinement. The software including source code is made available under a free software license.

## Abstract 2

A program for the digitization of Guinier powder diffraction images is described, which works with images from both optical and laser scanners. Thus, processing of data from storage-phosphor-based imaging plates and Ag-based photographic films is possible.

## 1. Introduction

Powder X-ray diffraction (XRD) is an important technique used for structural analysis of polycrystalline materials (Louër, 1998 ▸) which cannot be obtained as single crystals and for characterizing phase transformations (Klimakow *et al.*, 2010 ▸). Often combined with other techniques such as solid-state NMR (Watts *et al.*, 2016 ▸; Li & Sun, 2017 ▸), powder diffraction is routinely used to perform phase identification, indexing (Boultif & Louër, 1991 ▸), structure solution (Poojary & Clearfield, 1997 ▸), Rietveld refinement (Rietveld, 1969 ▸), quantitative phase analysis (Bish & Howard, 1988 ▸), and investigations of defects and disorder (Saleki-Gerhardt *et al.*, 1994 ▸) on a variety of different organic and inorganic crystalline materials (Harris *et al.*, 2001 ▸).

There are different diffractometers and camera methods used in powder X-ray diffractometry, which employ different geometries, X-ray optics and detection techniques (Jenkins, 2001 ▸). The Guinier camera (Pouget *et al.*, 2019 ▸), originally built by A. Guinier (1937 ▸), uses a beam-focusing Johansson monochromator (Johann, 1931 ▸) between the X-ray source and specimen (Rudman, 1967 ▸). Guinier cameras can be set up in different configurations, such as symmetric/asymmetric transmission and back reflection (Rudman, 1967 ▸). Owing to its good focusing capabilities providing sharp reflections on the focus cylinder and fast data acquisition (Ersson, 1979 ▸), the Guinier method, similar to others like the Debye–Scherrer or Bragg–Brentano methods, has never lost its importance for routine fast phase analyses. Moreover, the Guinier camera has a very simple setup which allows for easy heating and cooling and for transporting to various facilities, and it requires only small sample quantities.

Regarding the means of detection, the Guinier method has undergone several modifications (de Wolff, 1948 ▸; Brown, 1970 ▸; Dachs & Knorr, 1972 ▸; Ståhl, 2000 ▸; Ihringer, 1982 ▸; Ihringer & Rottger, 1993 ▸). Imaging plate (IP) detection was an upgrade from the conventional film detection and was introduced by Fuji Co. Ltd in the field of medical radiography in 1985 (Kato *et al.*, 1985 ▸). The IP method is based on the scanning laser stimulated luminescence (Sonoda *et al.*, 1983 ▸) of a photostimulable phosphor system such as BaF*X*:Eu^2+^ (*X* = B, I) (von Seggern, 1999 ▸). Since the late 1980s, this detection method has been adopted in synchrotron (Amemiya, 1995 ▸) and conventional X-ray crystallography (Kamiya & Iwasaki, 1995 ▸), such as usage in Guinier cameras (Amemiya & Miyahara, 1988 ▸; Gal *et al.*, 2005 ▸), and also as a radiation detector in other areas of physics (Izumi *et al.*, 2006 ▸). The advantages of using IPs in crystallography are (i) simultaneous detection of the entire diffracted beam interval, (ii) relatively good detection linearity (Sunghwan *et al.*, 2004 ▸), (iii) high lateral resolution down to 10 µm and (iv) a high dynamic range (Thoms, 1997 ▸) of up to six decades on commercial laser scanners.

The process of using IPs in radiology and crystallography consists of the following steps: X-ray exposure, laser scanning of the IP and erasing of the residual phosphor sites for reuse (von Seggern, 1999 ▸). X-ray photons are absorbed by lanthanide-activated inorganic phosphors such as BaF*X*:Eu^2+^ (*X* = Br, I) whereby electron–hole pairs are created (Takahashi *et al.*, 1984 ▸). Some of the produced electrons are trapped in color centers and can be ‘untrapped’ by irradiation with light with a long wavelength (*e.g.* red laser), after which the excited electrons recombine with the holes and emit light with a short wavelength (*e.g.* blue light), thus making it easy for them to be detected by a photomultiplier (Nanto, 2018 ▸). Unfortunately there seems to be no full agreement on the exact mechanism (Nanto, 2018 ▸; Schweizer, 2001 ▸; Wang & Riesen, 2015 ▸).

The ideal application in X-ray diffraction is the integration of an IP and a laser scanner into the diffraction camera. Such internal scanners give high-quality X-ray diffractograms. Unfortunately, upgrades of old X-ray diffraction cameras which have been developed for Ag-based chemical photography are not available. An alternative is to use such a storage-phosphor-based IP inside a ‘traditional’ diffraction camera and combine that with an external laser scanner, which has been used in many laboratories for decades (Gal *et al.*, 2005 ▸). In this way it is straightforward to re-enable old Laue, Guinier or Debye–Scherrer cameras, avoiding dark-room processing of the Ag-based films.

The result of the scanning process, depending on the camera type, is a grayscale bitmap which needs to be converted into plots of intensity versus scattering angle(s). For powder data digitization and reduction, there exist a number of software tools such as *FIT2D* (Hammersley, 2016 ▸), *powder3D* (Hinrichsen *et al.*, 2006 ▸), *SMC* (Davies, 2006 ▸), *MAUD* (Lutterotti *et al.*, 2007 ▸), *Datasqueeze* (https://www.physics.upenn.edu/~heiney/datasqueeze/index.html), *DIOPTAS* (Prescher & Prakapenka, 2015 ▸) and more (Rodriguez-Navarro, 2006 ▸). Although a program has been written in C++ for a similar purpose for Debye–Scherrer and Gandolfi-type diffraction patterns (Petrus *et al.*, 2012 ▸), to the best of our knowledge a software tool does not exist which converts scanned Guinier-type images into data columns (.xy and .raw formats) of angles and intensities and which allows for a convenient calibration of the angular scale.

The purpose of this study is to investigate the reliability of diffraction data from a ‘traditional’ Guinier camera upgraded with an external scanner and a respective image data conversion program.

## 2. Experimental details

### 2.1. Materials

The reference compounds SiO_2_, CaWO_4_ and Si crystallize in the low-quartz (*P*3_2_21), scheelite (*I*4_1_/*a*) and diamond structures ( 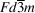 ), respectively. LaB_6_ (99.9%) was purchased from Smart-Elements GmbH (Austria) and was used as received. NaCl (min. 99%) was purchased from Chemsolute (Germany) and was used without further purification but was vacuum dried for quantitative powder XRD. Sodalite was synthesized (Jaeger, 1929 ▸) using a solid-state route from kaolin (Sigma–Aldrich), NaCl (min. 99%, Chemsolute) and Na_2_CO_3_ (min. 99%, Riedel-de Haën, Germany). Kaolin was first activated by heating at 873 K for 10 h, and Na_2_CO_3_ was heated at 523 K for 2 h in an oven in air. Stoichiometric amounts of kaolin, NaCl and Na_2_CO_3_ were turned into a paste by adding an appropriate amount of acetone and ball milled at 30 Hz for 20 min. The dried mixture was pressed into a pill (13 mm cell, 5 Mg load, 15 min) and heated with a heating ramp of 1 K min^−1^ at 1123 K for 24 h in air. After heating, the surface of the pill turned slightly red, probably due to iron impurity which was pushed to the surface during the long thermal treatment, while the inner part was white. XRD of the white powder showed good crystallinity, and ^23^Na and ^27^Al solid-state magic-angle spinning NMR (not reported here) proved that there was no amorphous side phase and no defect sites involving the two elements.

### 2.2. Powder XRD measurements

All powder XRD measurements were carried out on a Huber Guinier powder camera G621 (Rimsting, Germany) in asymmetric transmission configuration with the X-ray tube operating at 35 mA tube current and 45 kV voltage (raw data: https://doi.org/10.25819/fodasi/6). A curved Ge(111) monochromator was used to focus the incident beam and monochromatize it to select only Cu *K*α_1_ radiation.

Mylar foils (1 or 10 µm thick) were mounted onto metallic or 3D-printed sample holders, whose surface had been polished to achieve a flat plane. A thin layer of grease was evenly spread out at slightly elevated temperatures (∼343 K) onto the Mylar foils upon which the powder samples were deposited. The XRD samples were prepared with one, three or five traces of powders, the exposure time was set to 5–25 min depending on the sample and type of camera insert used, and the diffracted photons were recorded on BaFX:Eu-based IP films. Only the data from the middle trace were used in all cases. During measurements, the samples were moved backwards and forwards perpendicular to the X-ray beam.

#### 2.2.1. 3D printing of sample holders

**Figure 1:**
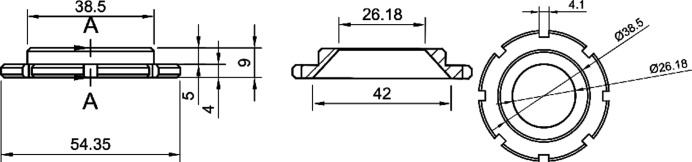
A technical sketch of the rotatable sample holder. All measurements are in millimetres.

A rotatable sample holder (Fig. 1 ▸) was printed with black acrylo­nitrile butadiene styrene filament manufactured by BASF. The 3D models were built with *FreeCAD* (Version 0.19; https://www.freecadweb.org/). The model was exported as an STL file (Amoureux & Pruski, 2008 ▸). The STL file was sliced with *Ultimaker Cura* (Version 4.10.1; https://ultimaker.com/software/ultimaker-cura) and then printed with a Prusa i3 MK3 3D printer using a 0.4 mm brass nozzle.

#### 2.2.2. Reading the IPs

The three types of IPs used in this study are BAS-IP MS 2040, BAS-IP TR 2040 and BAS-IP MS 2040 (FujiFilm). They consist of a polymer/storage-phosphor layer (BaFBr_1−*x*_I_*x*_:Eu^II^) and a magnetic backing. The ‘super resolution’ (SR) and ‘tritium screen’ (TR) IPs contain a blue dye to increase the readout resolution (‘anti-bleeding’), while the ‘multipurpose use’ (MS) IP does not and appears colorless. The TR type has a phosphor layer which, according to the manufacturer’s documentation, is thinner than that of the other IPs and is not protected with an organic polymer against water/humidity as the MS and SR type are.

The films were cut to the standard sizes of the Huber G621 camera using the original tools required for Ag-based photographic films. In order to study the decay of the latent images after X-ray exposure, the delay times before scanning were varied (the intensity loss of the scanned image as a function of delay time is plotted in Fig. S1 in the supporting information). Prior to the measurements, the IPs were erased for 10–15 s under a 500 W halogen lamp. These conditions were tested to be sufficiently long to guarantee complete erasure of the image on the IP. The X-ray-exposed IPs were kept in the dark and transferred into the IP holder of the Typhoon FLA 7000 scanner (GE Company, USA). They were read out in ‘phosphor imaging mode’ using a 650 nm laser. The delay between the end of the exposure and the start of scanning was around 1 min or less, and the scanning of the entire pattern took around 4 min. All the recorded diffraction images were digitized using the *IPreader* software introduced here. Averaging multiple measurements as described below was done by a tcl script which uses a linear interpolation of the intensities onto a common 2θ scale.

### 2.3. *IPreader* software

In the Typhoon FLA 7000 scanner used in this study the scanning resolution of 25 µm, in combination with the film length, yields an angular resolution of about 0.012° on the 2θ scale and a dynamic range for the grayscale of 16 bits (around five decades), which is sufficient for most applications in powder X-ray diffraction. The result of the scanning process is a bitmap image file which cannot be read directly by standard XRD software. The purpose of the *IPreader* software is to convert this image file into an XY ASCII file which includes intensity values *I* and the 2θ scale angles. The IP scanner saves the data in a bitmap file format which encodes the pixels with a grayscale of 16 or 32 bits in order to achieve the above-mentioned high dynamic range. The exported gel files from the scanner used in this study are a variant of TIFF but compress signal intensities by a ‘square-root compression’ and can be read by libraries and programs which can read TIFF files such as the program *ImageJ* (https://imagej.nih.gov/ij/; Schneider *et al.*, 2012 ▸). The Typhoon scanner can also export data on a linear *y* scale in the form of 16-bit TIFF files. When cutting TIFF/gel images of this kind it is important to use software which does not automatically downgrade to 8-bit file formats or information will be lost.

**Figure 2:**
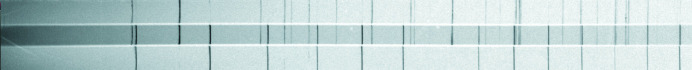
Images of powder XRD patterns of (top) low-quartz SiO_2_, (middle) LaB_6_/SiO_2_ and (bottom) LaB_6_ recorded with Cu *K*α_1_ radiation on a Huber G621 Guinier camera equipped with a BAS-IP TR 2040 IP.

A typical image of a diffraction experiment contains the diffractograms of three different samples (Fig. 2 ▸). For geometric reasons the highest resolution is achieved in the middle trace. Therefore only data from the sample of interest mounted on the middle trace (alone or in combination with a reference compound, *e.g.* SiO_2_ or Si) are used in this contribution (Fig. 2 ▸). A small offset and scale error (typically <0.1°) needs to be tolerated if either the bottom or top sample traces are used to calibrate the scale of the middle trace.

**Figure 3:**
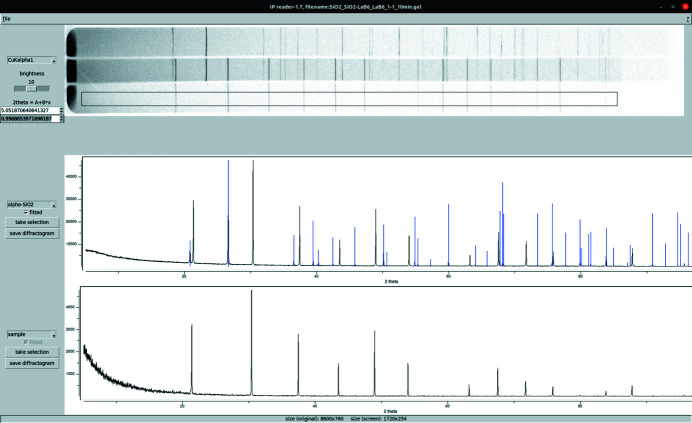
The user interface of the *IPreader* software (Version 1.7). A scanned image is loaded (upper image, three traces), and digitized patterns are shown for (middle) the SiO_2_/LaB_6_ mixture and (bottom) pure LaB_6_ of the selected slice. The top diffractogram was obtained from the middle sample trace, and the bottom diffractogram from the bottom sample trace. Calibration of the 2θ axis was done from the SiO_2_ reflections (blue lines) using the manual mode (see text).

The *IPreader* software is written in tcltk (Version 8.6) to achieve a user-friendly graphical user interface which is portable to different operating systems. The ‘gel’ or ‘unwrapped’ false color image files of the scanner are read internally by making function calls to the library *LibTIFF* (http://www.libtiff.org/). This converts the grayscale values inside a selection box into a vector of integer values, which is triggered by pressing the button ‘take selection’ (Fig. 3 ▸). A standard deviation of the intensity values is determined from the variation of intensities within a column of the image. A typical conversion process requires calibration of the 2θ scale by selecting the trace and applying ‘take selection’. The chosen radiation and reference compound need to be manually selected from a drop-down menu. If no reference material is used to calibrate the scale (see below), then the sample traces can still be digitized, in which case the 2θ values will start at an angle of 0°. The greater the size of the boxes in the vertical direction, the more pixels corresponding to the same 2θ value will be averaged and the better the final signal-to-noise ratio. On the other hand, the resolution is usually better, especially in the low-angle region, if only a smaller height of the image is used.

After setting up the reference trace, the user needs to shift the selection box from the reference trace to the middle trace referring to the compound of interest. Keeping the selection box makes the software keep the same scale for the diffraction angles.

During the handling of the reference trace as described above, the software performs a peak picking process and automatically sets the 2θ scale. This is achieved by assigning the positions of the reflections of the reference trace to the internally stored *d* values of the reference compound and then performing a linear regression from which a linear function is determined which converts lateral positions into 2θ angles. The result of the automatic peak assignment is visualized with the help of the blue lines appearing in the reference diffractogram after selecting the reference compound from the pull-down menu. The peak assignment (and thus 2θ calibration) is successful when the positions of the blue lines agree with the positions of the experimental maxima (black curve). The computed scale with its parameters *A* and *B* is directly applied to all diffractograms and is used to compute the 2θ values which are stored in the XY file upon data export. If the automatic peak assignment is not successful it is possible to set the scale parameters *A* and *B* manually. If afterwards the checkbox ‘fitted’ is activated, then the scale will be set from the peaks closest to the current blue lines, again making use of a linear regression. The latter approach is more robust than the automatic peak assignment. The software has been used at the University of Siegen for several years and tested with diffractograms of different signal-to-noise ratios obtained with Cu *K*α_1_ radiation.

The software is published under a GPL and is made available via the github repository (https://github.com/storkan/IPreader). Together with the software, a script is distributed which allows the averaging of XY files. The challenge that two scanned diffractograms will not have precisely the same 2θ angles is overcome by a linear data interpolation scheme.

### 2.4. Rietveld refinement

Rietveld refinement (Rietveld, 1969 ▸) and other types of line shape analyses were performed using the *TOPAS-Academic* software package (Version 7; Coelho, 2018 ▸).

## 3. Results and discussion

Diffractograms of several compounds, *i.e.* LaB_6_, CaWO_4_, Si, and a mixture of sodalite and NaCl, were recorded on a Huber Guinier camera using IPs and an external IP scanner in order to test the digitization procedure. To establish the quality of the digitized diffractograms, the diffractograms of LaB_6_, Si and the sodalite–NaCl mixture were analyzed using Rietveld refinements. The difference plots were checked for errors which could result from the measurements and digitization procedure, for example profile distortions due to nonlinear stretching of the IP. Furthermore, it was tested whether the angular scale is stable enough to improve the signal-to-noise ratio through averaging of multiple diffractograms for the example of CaWO_4_. The *IPreader* software is described in more detail in the *Experimental details* [Sec sec2] section above.

### 3.1. General considerations

The exposed IPs are light sensitive. For this reason the opening of the camera and mounting of the IP on the holder in the laser scanner require a dark environment. In comparison to the natural decay times of latent images stored in the storage phosphor, the handling period (<5 min) for obtaining a digital image is short enough to neglect any loss of the signal intensity caused by the natural thermal decay of the trapped excited states (Fig. S1), which shows a bi-exponential decay with a short time constant of about 2–3 h and a long time constant >300 h. Upon testing the different types of IPs for the obtainable resolution on the diffraction signals of LaB_6_ (Fig. S2), we observed a small advantage of the TR and SR IPs over the MS type which is only relevant in the case of very sharp reflections of a compound. The higher resolution comes at the expense of slightly reduced readout intensities. For standard applications, all three types of IPs work equally well. The most critical degradation of the films we observed in the use of the IPs over several years is mechanical damage to the IP surface, which leads to intensity fluctations (both positive and negative) in the readout intensity.

An interesting application of the *IPreader* software is the digitization of old photographic films using a standard image scanner with a film holder for negatives operating with transmitted light. Suitable scanners can deliver 16-bit TIFF images which are compatible with the *IPreader* software, thus allowing the digitization of archived diffractograms stored in photographic films.

### 3.2. Rietveld refinement

LaB_6_ was used to determine the instrumental parameters of the Guinier camera, including any nonlinear angular deviations. The compound LaB_6_ provides diffractograms with very sharp reflections which are thus very sensitive to angular errors.

To improve the quality of the diffraction data, the following procedure was applied. Recording LaB_6_ data on powder traces while the sample was undergoing lateral motion resulted in a Rietveld refinement with relatively high *R*_wp_ values (>8%). Measurements performed on a series of LaB_6_ samples showed random changes in the relative intensities from one sample to the next. Therefore several measurements were averaged.

In the Rietveld refinement of LaB_6_ (99.9% purity), only a very small amount of strain-related broadening was taken into account (Table 1 ▸). The cell parameters were refined and compared with the NIST certified value for LaB_6_. The isotropic displacement factors were not refined but taken from a reference data set [International Centre for Diffraction Data (ICSD; https://icsd.fiz-karlsruhe.de/index.xhtml) collection code 152466]. From a series of variable thickness measurements (not reported here) it was empirically determined that diffraction patterns in the asymmetric transmission Guinier method are hardly affected by X-ray absorption, and thus no absorption corrections were included in the refinement (Cullity, 1978 ▸).

Asymmetric broadening arising from diffraction ring curvature upon digitizing using *IPreader* was minimized by selecting only a fraction of the full height of the recorded pattern. A linear 2θ offset function was required in the whole-profile pattern fit to remedy the nonlinear angular shift caused by stretching of the flexible IP or by its potential mispositioning. For the sharp reflections of LaB_6_, a nonlinear second- and third-order polynomial function further improved the fit to a small degree, and thus the third-order polynomial function was used. Both axial and equatorial divergence were refined, along with tube tails to account for the residual broadening due to the X-ray emission profile (despite the calibration of the monochromator, a tail on the right-hand side of the incident beam remained). Also refined were sample displacement and sample tilt. See Table S2 for all the refined parameters.

The fundamental parameters approach (FPA) (Klug & Alexander, 1974 ▸) was considered appropriate for the Guinier method, so no existing aberration models were applied except for the simple axial model as implemented in *TOPAS*. The lowest value of *R*_p_ achieved in the structure refinement of a pattern averaged over 24 measurements was 5.2% (Fig. S3).

Compared with a commercial state-of-the-art Guinier camera (Huber G670) which has an internal laser scanner, the old Huber G621 powder camera used with an external scanner as employed in this study has the disadvantage of the 2θ scale being less precise, which has an impact on the errors of the lattice parameters output by the Rietveld refinements. The differences are, however, not substantial (see below) and are often not visible when the width of the reflections is not as sharp as it is for LaB_6_. Using an internal reference, *i.e.* mixing the sample of interest and the reference, can alleviate this problem because then the nonlinear scale function can be fitted to the reflections of the reference compound within *TOPAS*.

### 3.3. Quantitative phase analysis

**Figure 4:**
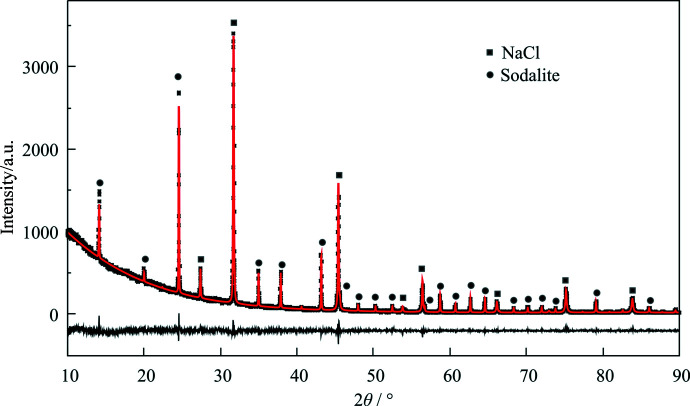
Rietveld analysis. Simulated curve (red solid line) and experimental pattern (small black points) measured on a mixture of NaCl (squares above reflections, mass ratio 58.5 ± 0.3%, refined ratio 60.0 ± 0.2%) and sodalite (circles above reflections, mass ratio 41.4 ± 0.3%, refined ratio 39.9 ± 0.2%), with the difference plot 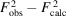 shown at the bottom (black solid line). The fitted red line was calculated using the fundamental parameters approach. Seven scans were measured on the same sample holder (*R*_p_ = 4.2%). The input file is provided in the supporting information.

A mixture of NaCl and sodalite was used without an internal standard to carry out a quantitative Rietveld analysis on data obtained on a Guinier camera (Fig. 4 ▸). Some measures were taken to reduce the uncertainties around data acquisition. Ground NaCl (min. 99% purity) and sodalite (purity expected to be more than 99%) were held in a vacuum for 20 h to remove the physisorbed water. The samples were briefly exposed to ambient atmosphere during weighing, but as soon as they had been thoroughly mixed in a sealed milling jar and deposited on the XRD sample holders they were protected by second layer of Mylar foil.

The Rietveld refinement was set up with the instrumental parameters from the calibration measurement of the LaB_6_ standard.

The Rietveld analysis of the mixture gave reasonable results (Table 1 ▸) with low residuals using a lattice strain model. Moreover, quantitatively the analysis provided a good agreement with the starting percentage values of the two components in the mixture. The experimental diffractogram in Fig. 4 ▸ was the average of seven scans on the same powder trace. The fit resulted in a residual *R*_p_ factor of 4.2%, and the discrepancy between the weighed and calculated values of NaCl and sodalite was tolerable compared with the range of expected values from the standard deviations.

### 3.4. Signal averaging

**Figure 5:**
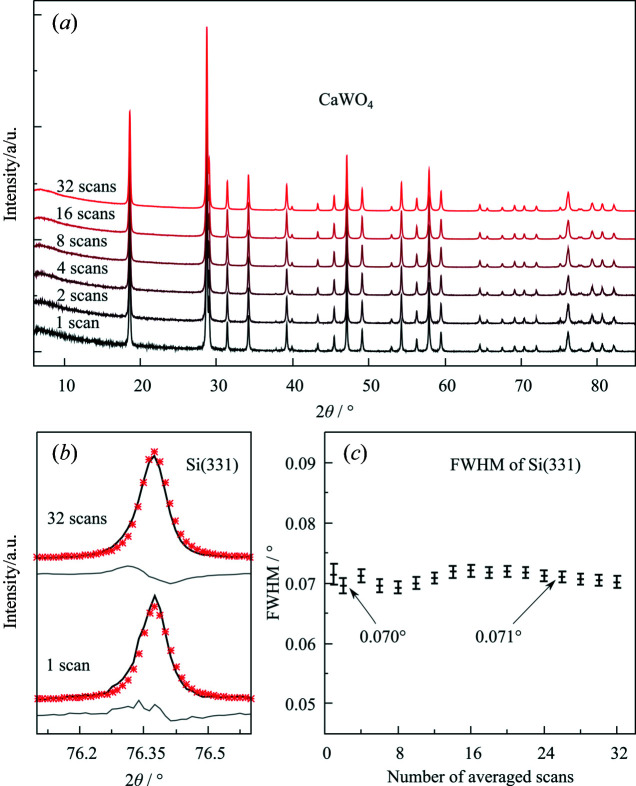
(*a*) Powder XRD diffractograms of CaWO_4_ of a single scan and of averaged diffractograms over 2, 4, 8, 16 and 32 scans (with each scan having been recorded for 10 min exposure time). (*b*) Si(331) reflection with a single scan (*R*_p_ = 7.1%, 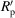 = 32.1%) and 32 scans (*R*_p_ = 4.3%, 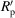 = 22.2%) averaged (solid black line) and their fitted data (red points). (*c*) FWHM values for the Si(331) reflection with various averaged scans up to 32.

Fig. 5 ▸(*a*) shows sequential averaging of XRD diffractograms of standard CaWO_4_ for up to 32 averaged scans. The averaging does not cause significant broadening of the reflections, as is apparent from the FWHM of the Si(331) reflection [Figs. 5 ▸(*b*) and 5 ▸(*c*)]. Signal averaging can therefore be a useful procedure to increase the signal-to-noise ratio.

## 4. Conclusions

The possibilities and limitations of upgrading a ‘traditional’ Guinier camera with an IP and an external laser scanner are demonstrated. The results indicate that the data quality, with an angular resolution of 0.012° on the 2θ scale, is sufficient for phase analysis, Rietveld refinement and averaging of powder patterns. Even structure solution may be possible if texture effects are eliminated. This can be achieved by averaging diffraction patterns from multiple measurements using a rotatable sample holder (see STL file in the supporting information).

The *IPreader* software which is published with this contribution allows for a simple calibration of the angular scale and conversion of the scanned pictures into different standard file formats. Small nonlinear errors of the 2θ scale may exist if an external reference is used.

It can be concluded that an upgrade with IPs and an external scanner may help to re-enable old diffractometers and provide extra wavelengths or measurement geometries at low cost. Moreover, a useful application of the *IPreader* software is the digitization of old photographic film diffractograms taken on Guinier cameras.

## Supplementary Material

Additional figures and TOPAS input files. DOI: 10.1107/S160057672200677X/vb5036sup1.pdf

Raw diffractograms, STL file of the sample holder for 3D printing: https://doi.org/10.25819/fodasi/6

## Figures and Tables

**Table 1:** Rietveld analysis parameters obtained for LaB_6_ and the mixture of polycrystalline NaCl and sodalite The Rietveld refinement was done using the FPA. The fitted parameters were the background (simulated with a Chebyshev polynomial with seven fittable parameters) and a preferred orientation according to March. Fixed parameters determined on an LaB_6_ reference compound were ‘tube tails’ (a simple axial model for describing the axial divergence of the beam) and Lorentz polarization.

	Calibrant	Quantified mixture	
Parameter	LaB_6_	NaCl	Sodalite
Data collection			
Wavelength (Å)	1.540562	1.540562	1.540562
Monochromator	Ge(111)	Ge(111)	Ge(111)
No of scans averaged†	Up to 24	7	7
Exposure time per scan (mins)	12	15	15
Digitized step size (°)	0.012	0.012	0.012
2θ range used in RR‡ (°)	10–90	10–90	10–90

Unit-cell and refined parameters			
Space group	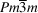	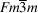	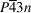
*a* (Å)	4.15685 (9)	5.63982 (2)	8.87640 (4)
Cell volume (Å^3^)	71.828	179.389 (3)	699.375 (12)
Density (g cm^−3^)	4.7108 (4)	2.1640	2.3012
Lattice strain ɛ (°)	0.000032 (2)	0.00435 (1)	0.00109 (1)

Residual factors			
*R*_p_, 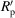 (%)	5.2, 16.9	4.2, 24.6	

Sample composition			
Purity of material (%)	99.9	>99	>99
Relative quantity weighed (%)		58.5 ± 0.3	41.5 ± 0.3
Relative quantity obtained from RR‡ (%)		60.0 ± 0.1	39.9 ± 0.1

† For LaB_6_, the sample was rotated by a few degrees after each exposure, while the mixture of NaCl and sodalite on the same sample holder was repeatedly irradiated keeping the same orientation of the sample holder.

‡ RR = Rietveld refinement.
