# Supplementary material for: Digitization of imaging plates from Guinier powder X-ray diffraction cameras
Source: J Appl Crystallogr. 2022 Aug 24;55(Pt 5):1097–103. doi: 10.1107/S160057672200677X (PMC9533741; doi:10.1107/S160057672200677X)
Supplement: Supplementary file 1 [file j-55-01097-sup1.pdf]

JOURNAL OF
APPLIED
CRYSTALLOGRAPHY

Volume 55 (2022)

Supporting information for article:

Digitization of imaging plates from Guinier powder X-ray diffraction cameras

Jamal Nasir, Nils Steinbrück, Ke Xu, Bernward Engelen and Jörn Schmedt auf der Günne

Supporting Information

Jamal Nasir[1], Nils Steinbrück[1], Ke Xu[1], Bernward Engelen[2], Jörn Schmedt auf der Günne[1]*

1 University of Siegen, Faculty IV: School of Science and Technology, Department of Chemistry and Biology, Inorganic materials chemistry and Center of Micro- and Nanochemistry and Engineering (Cμ), Adolf-Reichwein-Straße 2, D-57076 Siegen, Germany

2 University of Siegen, Faculty IV: School of Science and Technology, Department of Chemistry and Biology, Inorganic Chemistry I, Adolf-Reichwein-Straße 2, D-57076 Siegen, Germany

Figure S1 Fading of the diffraction signal on the imaging plate with time

Figure S2 Powder diffraction patterns of LaB_6 for the different types of imaging plates

Figure S3 Rietveld refinement for LaB_6

Table S1 Topas input file for LaB_6 refinement

Table S2 Topas input file for Rietveld refinement of a mixture of NaCl and sodalite

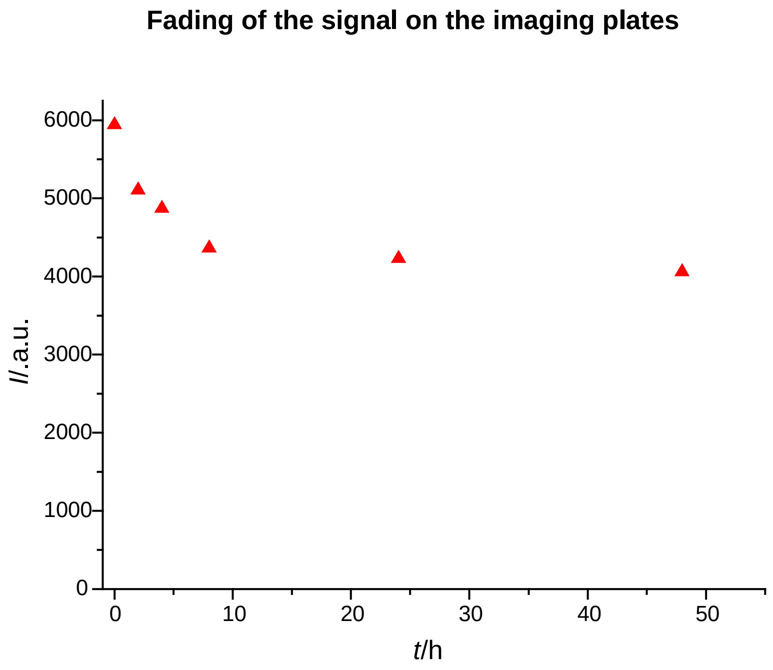

Figure S1 Fading of the signal on the imaging plate BAS-IP MS 2040. The most intense peak was measured as a function of the time delay between exposure of the imaging plate in the Guinier camera with $CuK_{\alpha 1}$ radiation and readout via the LASER scanner.

Powder diffraction patterns of LaB_6 for the different types of imaging plates

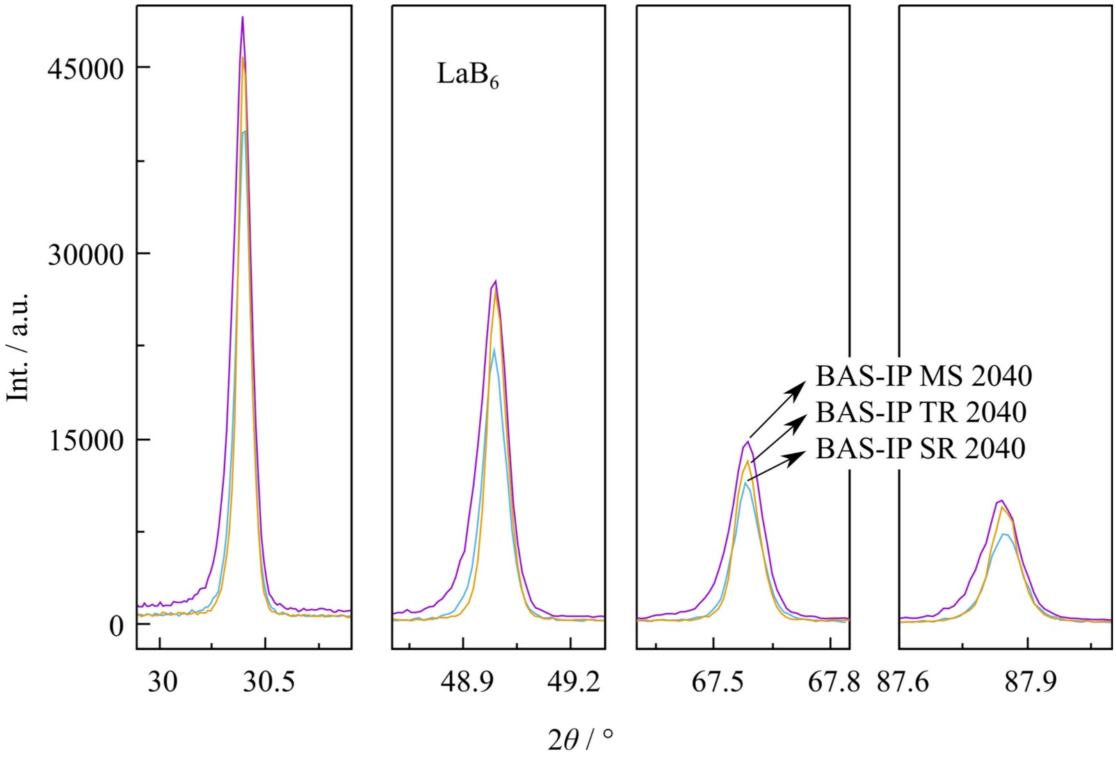

Figure S2 Powder X-ray diffraction data measured on LaB_6 with $CuK_{\alpha1}$ radiation in Guinier geometry using the same exposure time, sample (LaB_6) and data processing for the different types of imaging plates.

Rietveld refinement for LaB_6

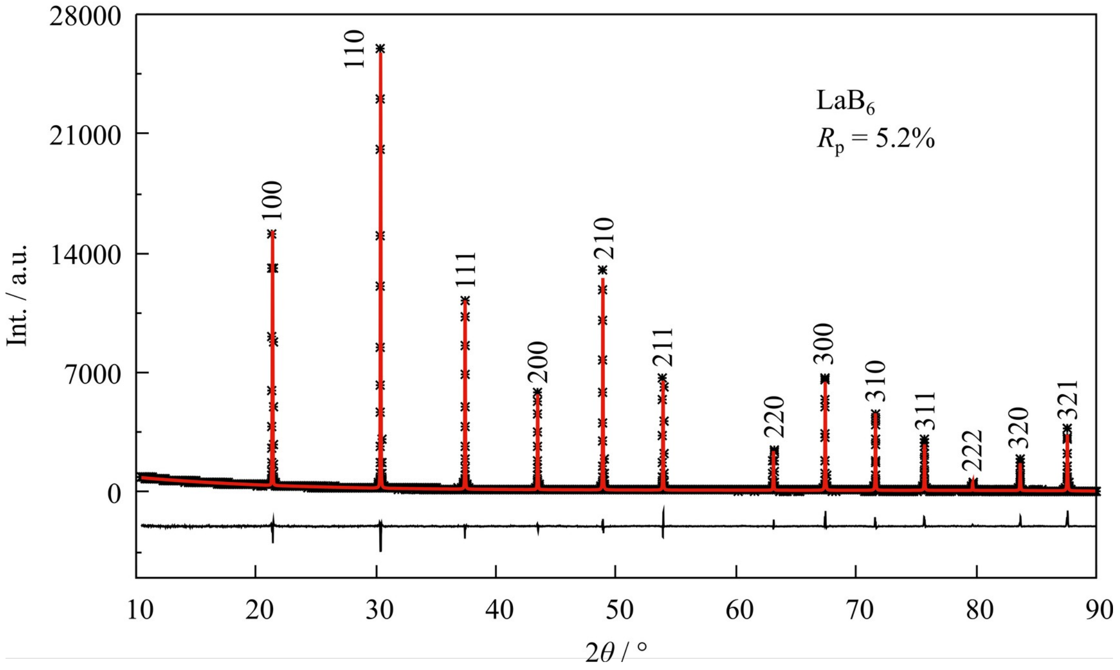

Figure S3 Rietveld refinement (red solid line) using Topas-Academic software on powder X-ray diffraction data (points) measured on LaB_6 in Guinier geometry and the difference plot $F^2_{obs} - F^2_{calc}$ (black solid line) to determine the instrumental parameters. 24 measurements were averaged using gnuplot1Daverage script. The refinement was done using fundamental parameters approach (FPA) and the lowest value of R_p achieved was 5.1%.

Topas input file for LaB_6 refinement

Table S1 Rietveld refinement input file (used in Topas academic package) with the refined parameters for standard LaB_6 powder diffraction (Figure S1) recorded on Guinier camera.

```
    r_p  5.23369805
    r_p_dash  16.9106426

    iters 100000
    chi2_convergence_criteria 0.001
    do_errors

xdd LaB6_24scans_averaged.xy

    x_calculation_step = Yobs_dx_at(Xo); convolution_step 4
    bkg @  264.361379`  0.396004983
            -293.075386`  0.711011265
            163.323794`  0.654621716
            -87.9493351`  0.584350288
            27.1413373`  0.542925493
            -11.0097319`  0.434766556
            -0.143938144`  0.379835129


   LP_Factor(!th2_monochromator, 27.28)                'd8 Ge(111) monochromator Vantec Cu Ka1
   CuKa1(0.0001)

   Specimen_Displacement(height, 0.189433714`  1.15769407)
   Specimen_Tilt(@, 0.0427354771`  0.000375919027)

   Divergence( 0.823576214_0.00574586197 )
   Simple_Axial_Model(!axial, 2.15331558_0.00498608309)
   Tube_Tails(, 0.00487350562,, -0.0388314018,, 0.00329872058_LIMIT_MIN_1e-05,,  0.0620669557)

    Rp 220
    Rs 57.3

'th2_offset

    prm a1  0.199234292`  0.16935602
    prm a2 -0.516894782`  1.26338315
    prm a3 -0.217338505`  0.0285479641
    prm a4  0.467382007`  2.31274557

    th2_offset = a1 Th^3 + a2 Th^2 + a3 Th^1 + a4;

    start_X 10
    finish_X 90

    Out_Yobs_Ycalc_and_Difference("Yob_Ycalc_Diff_LaB6_24scans_averaged .txt")
    Out_X_Yobs("2th_Yob_LaB6_LaB6_24scans_averaged .txt")

str
    phase_name LaB6
    space_group "P m -3 m"
    Cubic (@  4.156891  0.000217)  'NIST: a = 4.156826
    volume  771.830
    site La1 num_posns 1   x = 0;          y = 0;     z = 0;      occ La 1    beq 0.641
    site B1  num_posns  6   x = 0.1991;   y = 0.5;  z = 0.5;    occ B 1     beq  0.1
```

```
    prm E0G  1.5e-05`
    prm E0L  2.47327719e-05`  2.89168054e-06
    prm arg_E0G = 360/Pi E0G;:  0.00171887339`  0
    prm arg_E0L = 360/Pi E0L;:  0.00283416689`  0.000331362182 min=0; max=0.001
    Strain_G( arg_E0G )
    Strain_L( arg_E0L )
    prm E0 = Voigt_FWHM_GL(E0G, E0L);:3.21162022e-05`  2.41677075e-06

    scale @  0.00252836026`  5.058e-06
    r_bragg  5.04120868
    Phase_Density_g_on_cm3( 4.71071604)
```

Topas input file for Rietveld refinement of a mixture of NaCl and sodalite

Table S2 Rietveld refinement input file (Topas academic package) with the refined parameters for a mixture of NaCl (58.52% (>99% purity)) and sodalite (41.47% (>99% purity)) powder diffraction recorded on a Guinier camera

```
    r_p  4.2509422
    r_p_dash  27.4566057
```

```
    iters 100000
    chi2_convergence_criteria 0.001
    'do_errors
```

```
xdd NaCl_sodalite.xy
    x_calculation_step = Yobs_dx_at(Xo); convolution_step 4
    bkg @ 225.594237`_0.222349617
          -356.282615`_0.408919813
           233.911487`_0.36946733
          -117.881505`_0.321090623
          45.7104333`_0.281964798
         -13.8852126`_0.202360762
         1.85170222`_0.146934254


   LP_Factor(!th2_monochromator, 27.28)          'd8 Ge(111) monochromator Vantec Cu Ka1
   CuKa1(0.0001)

   Specimen_Displacement(height, -0.0158138078`_3.07078914)
   Specimen_Tilt(@, 0.0555516165`_0.00125843345)

   Divergence( 0.823576214_0.00574586197 )
   Simple_Axial_Model(!axial, 2.15331558_0.00498608309)
   Tube_Tails(, 0.00487350562,, -0.0388314018,, 0.00329872058_LIMIT_MIN_1e-05,,  0.0620669557)

    Rp 220
    Rs 57.3
```

```
'th2_offset

    prm a1  -0.0460899637`_0.411776407
    prm a2 0.0852462375`_3.30172676
    prm a3 -0.354627436`_0.0573505913
    prm a4  0.0720562818`_6.13690332

    th2_offset = a1 Th^3 + a2 Th^2 + a3 Th^1 + a4;
```

```
    start_X 10
    finish_X 90
```

```
    Out_Yobs_Ycalc_and_Difference("Yob_Ycalc_Diff_NaCl_sodalite .txt")
    Out_X_Yobs("2th_Yob_NaCl_sodalite .txt")
```

```
str
    phase_name Sodalite
    weight_percent  39.944`_0.144
    space_group "P -4 3 n"
    Cubic ( @ 8.876681`_0.000065)
    volume   699.442`_0.015
    site Al1  num_posns  6   x = 0.25;     y = 0;       z = 0.5;      occ Al+3 1.     beq  0.6
    site Si1  num_posns  6   x = 0.25;     y = 0.5;     z = 0;        occ Si+4 1.     beq  0.8
    site Na1 num_posns  8   x = 0.1785;  y = 0.1785;  z = 0.1785;  occ Na+1 1.     beq  1.7
    site Cl1  num_posns  2   x = 0;        y = 0;       z = 0;        occ Cl-1 1.     beq  2.3
```

```
    site O1   num_posns  24 x = 0.1399;  y = 0.1496;  z = 0.4382;  occ O-2  1.       beq  0.9



      prm E0G1  0.000690468452`
      prm E0L1  0.000636268743`  1.53099412e-05
      prm arg  E0G1 = 360/Pi E0G1;:0.0791218564`  0
      prm arg  E0L1 = 360/Pi E0L1;:0.0729110272`  0.00175439003 min=0; max=1
      Strain  G( arg  E0G1 )
      Strain  L( arg  E0L1 )
      prm E01 = Voigt  FWHM  GL(E0G1, E0L1);:0.00109387938`  1.09146675e-05

    scale @  2.09780981e-05`  1.055e-07
   r_bragg  2.47787372
   Phase_Density_g_on_cm3( 2.30099848`  5.05015659e-05)
str
    phase_name NaCl
    weight  percent  60.056`  0.144
    space_group "F m -3 m"
    Cubic ( @ 5.639567  0.000443)
    volume   179.389
    site Na1 num_posns  4   x = 0;        y = 0;       z = 0;      occ Na+1 1        beq  2.19235
    site Cl1 num_posns  4   x  = 0.5;      y = 0.5;      z  = 0.5;   occ Cl-1 1        beq  1.25933



      prm E0G2  0.00326776351`
      prm E0L2  0.00181470156`  1.36099672e-05
      prm arg  E0G2 = 360/Pi E0G2;:0.374458115`  0
      prm arg  E0L2 = 360/Pi E0L2;:0.207949481`  0.00155958736 min=0; max=1
      Strain  G( arg  E0G2 )
      Strain  L( arg  E0L2 )
      prm E02 = Voigt  FWHM  GL(E0G2, E0L2);:0.00435523301`  8.96682924e-06

    scale @  0.000509924394`  1.669e-06
    r_bragg  1.96360951
    Phase_Density_g_on_cm3( 2.16393042)
```
